# Visible light photonic integrated Brillouin laser

**DOI:** 10.1038/s41467-021-24926-8

**Published:** 2021-08-03

**Authors:** Nitesh Chauhan, Andrei Isichenko, Kaikai Liu, Jiawei Wang, Qiancheng Zhao, Ryan O. Behunin, Peter T. Rakich, Andrew M. Jayich, C. Fertig, C. W. Hoyt, Daniel J. Blumenthal

**Affiliations:** 1 Department of Electrical and Computer Engineering, University of California Santa Barbara, Santa Barbara, CA USA; 2 Department of Applied Physics and Materials Science, Northern Arizona University, Flagstaff, AZ USA; 3 Center for Materials Interfaces in Research and Applications, Northern Arizona University, Flagstaff, AZ USA; 4 Department of Applied Physics, Yale University, New Haven, CT USA; 5 Department Physics, University of California Santa Barbara, Santa Barbara, CA USA; 6 Honeywell International, Plymouth, MN USA

**Keywords:** Integrated optics, Lasers, LEDs and light sources, Micro-optics

## Abstract

Narrow linewidth visible light lasers are critical for atomic, molecular and optical (AMO) physics including atomic clocks, quantum computing, atomic and molecular spectroscopy, and sensing. Stimulated Brillouin scattering (SBS) is a promising approach to realize highly coherent on-chip visible light laser emission. Here we report demonstration of a visible light photonic integrated Brillouin laser, with emission at 674 nm, a 14.7 mW optical threshold, corresponding to a threshold density of 4.92 mW μm^−2^, and a 269 Hz linewidth. Significant advances in visible light silicon nitride/silica all-waveguide resonators are achieved to overcome barriers to SBS in the visible, including 1 dB/meter waveguide losses, 55.4 million quality factor (Q), and measurement of the 25.110 GHz Stokes frequency shift and 290 MHz gain bandwidth. This advancement in integrated ultra-narrow linewidth visible wavelength SBS lasers opens the door to compact quantum and atomic systems and implementation of increasingly complex AMO based physics and experiments.

## Abstract (web summary)

In this work the authors demonstrate on-chip integration of Brillouin lasing operating at visible wavelengths, with engineered design for stable output. This technical and scientific advance will help develop integrated light sources for quantum computing or atomic and molecular spectroscopy.

## Introduction

Ultra-narrow linewidth visible light lasers provide the spectral purity required for precision atomic, molecular and optical (AMO) physics including atomic clocks^1,2^, atomic and molecular spectroscopy^3–5^, and quantum sensing^1,6,7^. Historically, it has been necessary to use macroscopic laser systems locked to vapor cells or large optical reference cavities to obtain the low phase noise and high frequency stability needed to address narrow optical clock transitions in atoms^8,9^. While providing state of the art performance^1,10,11^, these lab-scale systems pose challenges for atomic and molecular experiments of ever-growing complexity and for a portable or even autonomous optical clocks. There is a need for visible wavelength lasers that are smaller and more reliable so that experiments can scale in the number of wavelengths, atoms or molecules, and complexity in general. Photonic integration provides a path to miniaturize these laser systems as well as improve their reliability^12–14^, reduce sensitivity to environmental disturbances, and enable systems with a larger number of entangled atoms^15,16^, higher sensitivity quantum sensors^6,17^, higher precision positioning, timing and navigation^18^, and probing of complex molecules^19–24^.

Stimulated Brillouin scattering (SBS) lasers, with their pump linewidth narrowing properties and ultra-low phase noise emission^25^ are a promising candidate for AMO physics and quantum applications. SBS emission in the visible has been achieved with fiber optic based resonators, exotic fiber, and bulk optic implementations^26–32^. Recently the coherence of a near infrared (NIR) fiber SBS laser was transferred to the visible to address the clock transition of strontium, however, this work required bulky, power inefficient, nonlinear frequency conversion^33^. To reduce system complexity and improve reliability, it is desirable to use a “direct-drive” approach, where the SBS laser directly emits at the desired visible wavelength, without intermediate conversion stages. Chip-scale SBS lasers operating in the NIR have exhibited impressive performance^34–41^, achieving sub-Hz fundamental linewidth^34^, 30 Hz integral linewidth over 100 ms, and 2 × 10^−13^ fractional frequency stability^42^. To date, visible light emission in a photonic integrated SBS laser has remained out of reach. This lack of progress has been primarily due to barriers such as realizing ultra-low loss Brillouin-active planar waveguides in the visible, leading to inefficient Brillouin scattering and preventing SBS lasing in the desired operating regime of long photon guiding lifetime, short phonon guiding lifetime, large resonator photon lifetime, and large resonator mode volume^34^. Overcoming these barriers, as well as realizing a visible light SBS laser in a wafer-scale integration platform, will lead to reduced size, cost and improve stability and robustness to environmental disturbances, and enable experiments and applications with increasing number of stable lasers and wavelengths.

**Fig. 1:**
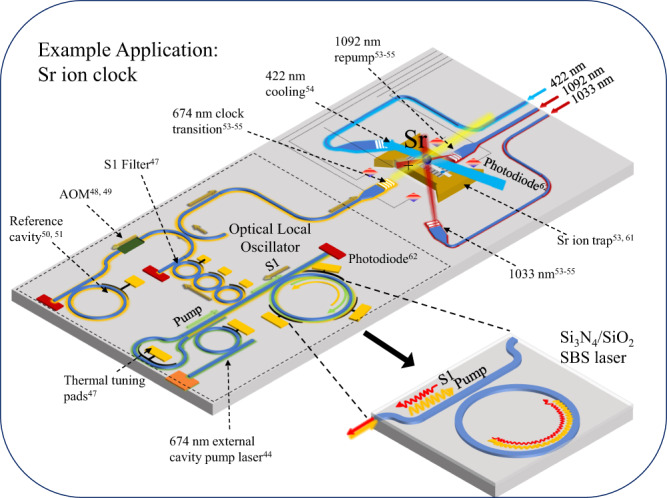
Example integration application of the visible light SBS laser. Example of how the integrated visible wavelength silicon nitride/silica waveguide stimulated Brillouin scattering (SBS) laser can be used as a clock transition optical laser oscillator (OLO) to lock to a strontium ion (^88^Sr^+^). A heterogeneously integrated 674 nm external cavity Si_3_N_4_ tunable laser can serve as the SBS pump, while a silicon nitride optical filter isolates and routes a portion of the SBS first order Stokes (S1) to an acousto-optic modulator (AOM) for sideband locking to an on-chip silicon nitride waveguide frequency reference cavity. The reference cavity stabilizes the SBS sideband laser for locking to the narrow atom transition. A single ^88^Sr^+^ ion trap is shown as example of chip-integrated trap^53,61^. Fiber-coupled 1092 nm and 1033 nm lasers can be converted to free-space beams for state re-pumping^53–55^ and on-chip photodiodes^62^ can provide monitoring for signal feedback for locking. The image on bottom right illustrates the reported Si_3_N_4_/SiO_2_ SBS laser.

In this work we report demonstration of visible light Brillouin lasing and visible light spontaneous Brillouin scattering in a photonic integrated waveguide structure. The laser is a ring-bus resonator design fabricated using a silicon nitride (Si_3_N_4_) core, oxide clad, moderately confining waveguide structure. Brillouin lasing at 674 nm is demonstrated with a 14.7 mW optical threshold, a 45% slope efficiency, and 9.28 mW on-chip output power for the first order Stokes (S1) with linewidth narrowing to 269 Hz as the pump power is increased from below threshold up to the second order Stokes (S2) emission threshold. This laser demonstration requires advances in waveguide loss reduction techniques including optimized geometry and process annealing to reduce top-side and side-wall scattering, and nitride surface and bulk oxide absorption^43^ at visible wavelengths. These advances enable record-low waveguide losses (~ 1 dB/m) and record-high Q (60 Million) at 674 nm, to the best of our knowledge, in a 2.68 × 10^4^ μm^3^ mode volume resonator. To measure visible light Brillouin gain and its Stokes frequency shift in a photonic waveguide, detection of the weak spontaneous Brillouin gain backscattered signal is enabled without the benefit of stimulated gain measurements. Traditional real-time pump-probe stimulated Brillouin gain measurements were difficult to perform due to the absence today of 26 GHz phase modulators that operate at 674 nm and 698 nm and 1348 nm semiconductor sources that can be frequency doubled without filtering out the sideband. To accurately predict the ~26 GHz Stokes frequency shift, we utilize a multi-physics simulation (details in the Methods section) and measure the Stokes shift by time averaged heterodyne Brillouin spectroscopy between the pump and backscattered signal which is guided. We measure a 25.110 GHz first order Stokes frequency shift and a 290 MHz gain bandwidth; accurately predicted by our simulations. We predict a peak Brillouin gain of 2.73 (W m)^−1^. The gain measurements guide the laser ring-bus resonator design, with a 3.587 GHz free-spectral range (FSR), equal to 1/7 of the 25.110 GHz S1 frequency shift. To highlight the versatility of this laser, we also demonstrate SBS lasing at 698 nm using the same waveguide materials and design, with mask-only changes. The 674 nm and 698 nm wavelengths are chosen to highlight applicability to ion and neutral strontium clock transitions respectively. The SBS laser waveguide is compatible with wafer-scale silicon nitride foundry processes and can be integrated with other photonic elements^19^. As an example, this laser can be used as a compact “direct-drive” optical laser oscillator (OLO) for a strontium ion clock (see Fig. 1). In this example, an integrated 674 nm semiconductor laser^44^ pumps the silicon nitride SBS laser to generate a reduced fundamental linewidth backward propagating first order Stokes wave (S1)^14,45,46^. The return Stokes signal, S1, can be filtered using an integration compatible silicon nitride waveguide dual-bus filter^47^. A tunable sideband, such as that generated by an acousto-optic modulator^48,49^ (AOM), is locked to a silicon nitride waveguide optical reference cavity^50,51^ to reduce the integral linewidth and provide the carrier stability needed to address the atomic optical clock transition. The stabilized 674 nm OLO can be coupled to a ^88^Sr^+^ ion in an electrostatic trap^33,52^ using, for example, silicon nitride waveguide to free-space grating couplers^53–55^. Additional cooling and repump beams can be provided via lasers coupled to silicon nitride waveguides and free-space grating couplers^54^.

## Results

### Visible wavelength SBS laser resonator

**Fig. 2:**
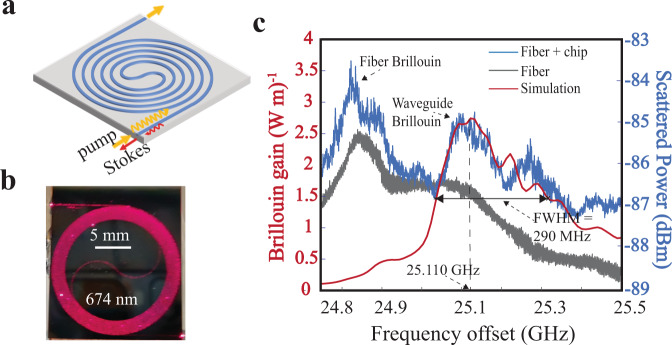
Waveguide design and 674 nm spontaneous Brillouin measurement. **a** Two meter single mode waveguide spiral used for the spontaneous Brillouin gain measurement. **b** Photograph of the actual 2 meter spiral shown while illuminated with 674 nm light. 5 mm scale is shown for reference. **c** Measured and simulated spontaneous Brillouin gain with 25.110 GHz first order Stokes (S1) frequency shift, 2.73 (W m)^−1^ gain peak, and 290 MHz bandwidth. The resolution bandwidth (RBW) of electrical spectrum analyzer (ESA) was set at 100 Hz. The measured blue curve shows the Brillouin contribution from both the fiber and silicon nitride waveguide, while the gray trace shows contribution from only the fiber, which confirms that the peak at 25.110 GHz is due to waveguide spontaneous Brillouin scattering.

The SBS laser resonator is based on an ultra-low loss single mode Si_3_N_4_ core and SiO_2_ cladding waveguide that is designed to operate at 674 nm. The waveguide consists of a 20 nm tall and 2.3 μm wide silicon nitride core deposited and etched on a lower thermally grown oxide cladding on a silicon substrate, with a TEOS-PECVD deposited upper cladding^47^ (cross section shown in Supplementary Fig. 4) and a final two-step anneal at 1050 °C for 7 h and 1150 °C for 2 h which is optimized anneal process for our waveguides. For further fabrication details see Methods. To maximize the spontaneous Brillouin signal, we fabricate a 2 meter on-chip spiral waveguide with ~ 1 dB/m loss and measure the small $\sim \!{10}^{-18}{W}$ (spectrum analyser resolution bandwidth of 100 Hz) spontaneous Brillouin signal (Fig. 2a, b).

Multi-physics vectorial simulations that incorporate actual measured materials and device parameters are used to predict the frequency offset and the Brillouin gain shape (red curve in Fig. 2c) (for details see the Methods and Supplementary Note 5). The weak back-scattered signal is measured by heterodyne detection of the pump-Brillouin beat note (Fig. 2c) using an ESA (for details see the Supplementary Note 2). Using time-averaged detection on an electrical spectrum analyzer (ESA) we measure a 25.110 GHz peak frequency shift and 290 MHz gain bandwidth, which agrees with our numerical simulations. The broad gain bandwidth and skewed line shape is due to the continuous generation of photons in the ultra-low loss optical waveguide without acoustic waveguiding, which permits coupling to a continuum of bulk acoustic phonon states within the waveguide oxide cladding^34,37^. The simulated Brillouin gain coefficient is 2.73 (W m)^−1^. Brillouin scattering in the optical fiber used to deliver the 674 nm pump laser light is distinguished from the waveguide Brillouin scattering (Fig. 2c blue curve) by decoupling the fiber from the chip and making an independent measurement (Fig. 2c gray curve), described further in the Supplementary Note 5).

**Fig. 3:**
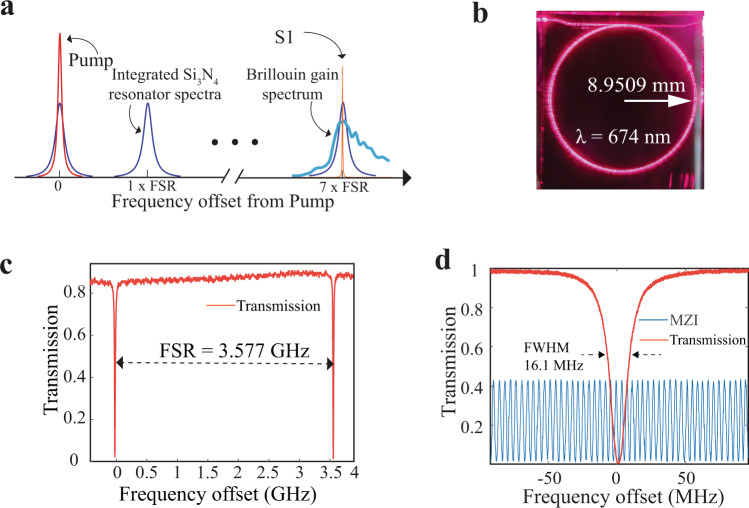
Stimulated Brillouin scattering (SBS) resonator design, FSR and quality factor. **a** Resonator design with free-spectral range (FSR) = 3.587 GHz equal to 1/7 of the 25.110 GHz spontaneous Brillouin peak gain frequency shift. **b** Photograph of silicon nitride waveguide resonator illuminated with fiber-coupled 674 nm laser light. The resonator radius is 8.9509 mm. **c** Measured resonator free-spectral range (FSR) = 3.577 GHz at 674 nm. **d** Quality factor (Q) measurement (red trace) performed using radio frequency (RF) calibrated unbalanced Mach–Zehnder interferometer (MZI) (blue trace). Full width half maximum (FWHM) linewidth = 16.1 MHz loaded Q = 27.7 million and intrinsic Q = 55.4 million and loaded Q = 27.7 million at 674 nm.

The SBS laser resonator is a 8.9509 mm radius bus-coupled ring structure with a free-spectral range (FSR) designed to be 1/7 of the measured 25.110 GHz peak Stokes shift at 674 nm (Fig. 3b and Fig. 3c) and a bus-to-ring power coupling coefficient^34^ к^2^ of ~ 1.5%. We design the ring to have multiple FSRs per Brillouin Stokes frequency shift to facilitate alignment of the Stokes shift and cavity resonance, increase the cavity volume, and provide robustness to fabrication variations^34^. A high intrinsic Q of 55.4 million and loaded *Q* = 27.7 million at 674 nm is achieved (Fig. 3d). These measurements are made using an RF calibrated Mach–Zehnder interferometer^47,56,57^ (MZI), and yield a low propagation loss of 1.09 dB m^−1^ at 674 nm (see Methods).

### Visible light 674 nm SBS lasing

**Fig. 4:**
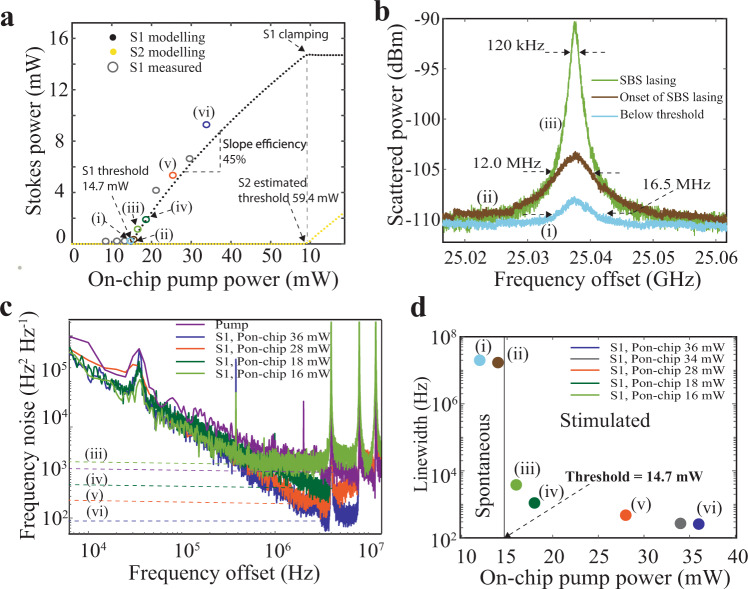
Stimulated Brillouin scattering (SBS) Stokes threshold, power and linewidth measurements. **a** Pump optical on-chip power (P_on-chip_) vs. first order Stokes (S1) signal power. Measured S1 laser threshold of 14.7 mW, corresponding to a threshold density of 4.92 mW μm^−2^, and a 45% slope efficiency. Modelling of the S1 optical power (black dots) accurately predicts the measured S1 optical power as the pump power is increased from below to above threshold. The predicted second order Stokes (S2) threshold is ~60 mW (yellow dots). **b** Measurement of S1 emission linewidth plotted on a logarithmic scale, below threshold (blue trace), just below threshold (brown trace) and just after threshold (green trace). The Brillouin emission linewidth evolves from a spontaneous dominated linewidth of 16.5 MHz, which is approximately the SBS gain filtered by the cold-cavity resonator linewidth ~16 MHz, to the onset of stimulated Brillouin, measuring a 12 MHz linewidth just below threshold, to a stimulated dominant 120 kHz linewidth just above threshold. The resolution bandwidth (RBW) of electrical spectrum analyzer (ESA) was set at 1 kHz. **c** Frequency noise measurements of S1 using a radio frequency (RF) calibrated fiber optic Mach–Zehnder interferometer (MZI) frequency discriminator. The S1 emission fundamental linewidth for each pump input condition shown in (**c**) is indicated by the corresponding horizontal dotted line. The free-running pump frequency noise (purple trace) is for an unlocked pump (i.e., not locked to the stable cavity or SBS resonator). As the pump is increased, a decrease in fundamental linewidth (curves ii–v) is measured. Since the back reflected pump is not optically filtered before frequency noise discrimination, there is a pump noise contribution to the measured frequency noise of S1. Just above threshold, the conversion from pump to S1 is low, and the white noise floor at 16 mW pump (green) is a combination of pump, S1 and their beat note. As the on-chip pump power (P_on-chip_) is increased, the intra-cavity S1 photon number increases while the pump signal is depleted and decreases to below 10 dB of the Stokes for all other FN traces. **d** Summary of beat note and fundamental linewidths from (**b**) to (**c**).

The SBS laser resonator is pumped by an off-chip tapered optical amplifier (TA) that is seeded with 674 nm light from a continuous wave external cavity diode laser (see experimental setup details in Supplementary Note 3). The TA output is coupled to the waveguide SBS resonator through a high-power fiber circulator. A maximum of 35 mW on-chip power is delivered to the waveguide bus, limited by a 180 mW TA maximum output power and ~4 dB fiber-to-facet coupling loss. The backward propagating S1 signal is measured using a 3-port fiber optic recirculator. The measured and simulated S1 powers are plotted vs. the pump power in Fig. 4a. A clear S1 threshold is observed for an on-chip pump power of 14.7 mW corresponding to a threshold density of 4.92 mW μm^−2^ and a 45% slope efficiency is measured, both in good agreement with our SBS model^56^ (for details of model see Supplementary Note 6).

In addition to verifying the laser threshold, we demonstrate a decrease in the S1 emission linewidth as the pump power is increased from below threshold, through threshold, and above threshold^34,57^. Below threshold, the optical power spectrum is measured using a heterodyne beat note produced by mixing the backward propagating S1 with the pump on an ESA (see Supplementary Note 3). To minimize the contribution of the pump linewidth to the measured beat note we lock the pump laser to a commercial high finesse ultra-stable cavity (Stable Laser Systems^TM^). Well below threshold (i), the scattered light is produced by uncorrelated spontaneous scattering from thermal phonons and is linearly filtered by the cavity resonance which is ~16.1 MHz. As threshold is approached, the spontaneous emission spectra, point (ii) in Fig. 4b, measures FWHM at 12.0 MHz, indicating the onset of stimulated emission, since the emission spectra is narrower than the cold-cavity resonance FWHM.

At just above threshold (iii), we see a dramatic 100× narrowing of the linewidth to 120 kHz as SBS dominates the emission (Fig. 4b, trace (iii)). At all points above threshold, we measure the frequency noise of S1 using an optical frequency discriminator (OFD) (see Methods and Supplementary Note 3). The fundamental linewidth ($\triangle \nu$) is defined^34,38^ as the far-from-carrier white frequency noise floor, in Hz^2^Hz^−1^, multiplied by $\pi$. In Fig. 4c the noise floor for each pump power input is indicated by horizontal dashed lines (iii–vi). As the pump power increases beyond S1 threshold, the fundamental linewidth drops dramatically from 1.1 kHz (iv) to 269.7 Hz (vi). These linewidth results are summarized in Fig. 4d, indicating the integral linewidths for points (i–ii) below threshold, and the fundamental linewidths for the frequency noise curves in (iii–vi) in Fig. 4c. We were not able to provide the required on-chip pump power, 59.4 mW, to achieve lasing of the second order Stokes (S2). Future work will look further into noise properties measured using stabilized pump sources and exploring linewidth behavior as S1 approaches the S2 lasing threshold.

### Demonstration of 698 nm SBS lasing

**Fig. 5:**
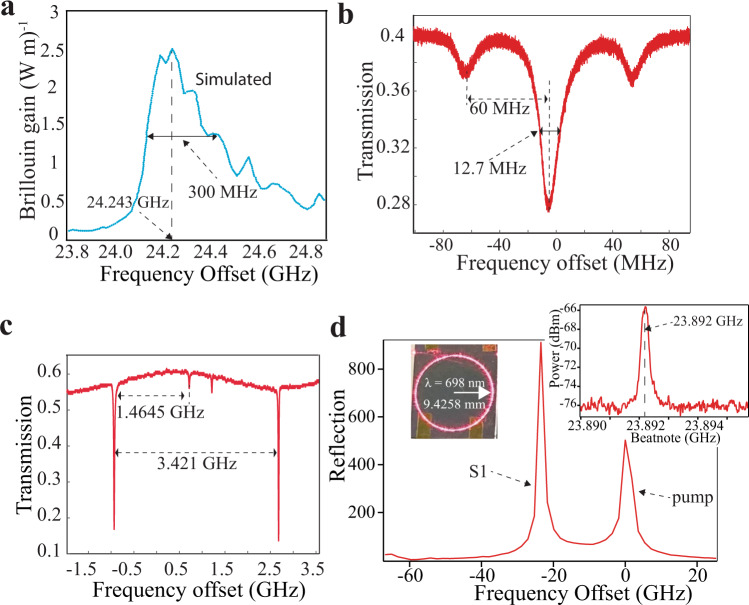
SBS lasing at 698 nm. **a** Multi-physics simulation of waveguide spontaneous Brillouin gain spectra for pump at 698 nm. Brillouin gain spectrum width of ~300 MHz and Stoke frequency shift of 24.243 GHz. **b** The measured full width half maximum (FWHM) resonator linewidth at 698 nm is 12.7 MHz and the measured intrinsic and loaded Qs are 60 million and 33.8 million respectively. **c** The resonator free-spectral range (FSR) at 698 nm is measured to be 3.421 GHz by applying sidebands at 1.4645 GHz. **d** First order Stokes (S1) and the pump measured on an optical spectrum analyzer (OSA), with inset showing the pump-S1 beat note measured at 23.892 GHz on an electric spectrum analyzer (ESA). Inset on left shows the 9.4 mm radius resonator fiber-coupled with 698 nm pump laser light.

We demonstrate the versatility of this SBS laser design by operation at another atom transition related visible wavelength. The silicon nitride bandgap supports low loss for wavelengths down to ~405 nm^13^, making this a powerful tool for a broad range of visible light atomic transitions. We design and fabricate a 698 nm SBS resonator using the same 674 nm waveguide design and geometry (as verified by optical mode simulations, see Supplementary Note 4) with modifications to the FSR and bus-ring coupling gap. At 698 nm, our multi-physics simulation predicts a 24.243 GHz Stokes shift and 300 MHz Brillouin gain bandwidth (Fig. 5a). 698 nm is chosen to match the neutral strontium atom clock transition. As with the 674 nm laser, the FSR is designed to be 1/7 of the S1 frequency shift, resulting in a 9.4 mm radius resonator design. A 3.4 μm bus-ring coupling gap is chosen to operate the resonator in the under-coupled regime with a power coupling coefficient of ~1%. For the fabricated devices, we measure a 12.7 MHz cavity resonance width, a 60 million intrinsic Q, a 33.8 million loaded Q, and a 3.421 GHz FSR (see Fig. 5b and Fig. 5c). Lasing at S1 is observed by pumping the cavity with a Ti:sapphire laser at the expected pump-Stokes frequency offset as measured shown in Fig. 5d. The observed pump is a reflection from the resonator far facet. A pump-S1 heterodyne 23.892 GHz beat note is measured as shown in Fig. 5d inset (see Supplementary Fig. 3). The 351 MHz beat note offset from our simulated shift and is most likely due the slight offset between the Brillouin gain peak and the narrow cavity resonance. The pump laser is free running (i.e., stabilized neither to the resonator nor a reference optical cavity), and the beat note drifts on the order of 100 kHz over tens of milliseconds. The on-chip pump power is 108 mW and the measured on-chip pump threshold power (P_th_) is ~75 mW.

## Discussion

We report demonstration of visible light SBS lasing and measurement of Brillouin gain in a waveguide photonic integrated circuit. The 674 nm laser is designed to serve as a low phase noise “direct-drive” chip-scale source that can couple directly to the ^88^Sr^+^ ion clock transition without the need for intermediate frequency translation and has wide application to other visible light ultra-low phase noise applications including quantum and precision metrology. The bus-coupled ring laser design produces Brillouin linewidth narrowing in the visible by operating in the regime of long photon lifetime, short phonon lifetime, large cavity volume, and long resonator decay time. To meet these lasing requirements, prior limitations are overcome and advances in visible light photonics required, including low waveguide ~1 dB/m losses and high resonator intrinsic 55.4 million Q, in a 2.68 × 10^4^ μm^3^ mode volume resonator. Since visible wavelength loss due to waveguide side-wall scattering is more severe at shorter wavelengths than in prior 1550 nm designs^34^, waveguides are designed by carefully reducing the optical mode overlap with the waveguide surface. With these advances, our laser operates at a 14.7 mW optical pump threshold with a 45% slope efficiency. Brillouin emission linewidth narrowing is demonstrated as the pump is increased from below threshold to above threshold, achieving 269.7 Hz fundamental linewidth at an on-chip pump power of 36 mW. Assuming the slope efficiency is constant and a calculated S2 threshold power of 59 mW (Fig. 4a), at the onset of S2 lasing (threshold), we estimate the S1 cavity photon number can be increased by a factor of 2× leading to an expected S1 linewidth of ~153 Hz^56^. Laser frequency noise sources include SBS fundamental noise^38^, intrinsic noise of the SBS cavity, noise coupling from backscattered pump, and technical noise sources in the pump laser and SBS resonator as well as pump amplitude to phase noise conversion. These noise sources can be decreased by locking the resonator, pump laser, or modulated S1 emission to an optical reference cavity^42^. Combining these advances with the results reported here shows promise for visible laser linewidth and stability for precision applications normally requiring table-top laser systems. Using this laser design, SBS lasing can be achieved at other visible wavelengths by making mask-only changes and a change in the pump source. To illustrate this versatility, we demonstrate lasing at 698 nm, a wavelength suitable to probe long-lived transitions in neutral strontium.

To improve SBS laser efficiency and further reduce linewidth, our simulations show that improvements can be made with closer FSR matching to the Brillouin gain peak shift. Calculations predict a 7 mW threshold as well as higher S1 optical output power. FSR optimization can be achieved by adjustment of the SBS cavity using waveguide tuning utilizing thermal^58^ and piezoelectric^59^ techniques. We predict that with the continued increase in S1 photon number up to the S2 threshold and with pump filtering, the fundamental linewidth can be reduced to ~2 Hz. Further improvements will require lowering the visible light loss, by exploring additional silica deposition processing techniques^60^ and different waveguide modes for lasing. Our side-wall scattering loss models^60^ suggest a significant improvement of 3×–4× is possible if the TM0 mode is used instead of TE0 mode. Such a loss reduction will linearly translate to a lower threshold and a smaller fundamental noise limit but will require careful design of couplers to suppress the TE0 mode to avoid crosstalk. Other possible improvements to further reduce the linewidth include modulating the laser resonator with a grating, to split the second order Stokes (S2) resonance and prevent S2 emission and further increase in S1 optical power^56^. Given the transparency and bandgap of the silicon nitride core and the low loss achievable down to ~405 nm, this platform can support a wide range of SBS photon-phonon interactions and as such, wavelengths for a variety atomic and molecular transitions. Future work will involve demonstrating this design across the broad range of silicon nitride waveguide transparency (e.g., Yb @ 578 nm, Ca + @ 729 nm and waveguides with higher bandgap that can support the UV (e.g., Al+ 267.4 nm).

## Methods

### Fabrication process

The fabrication process is foundry compatible and starts with 15 µm-thick thermal oxide that is grown on a 100 mm (4 inch) diameter, 1 mm thick silicon wafer substrate to form the lower cladding. The waveguide layer is formed by depositing 20 nm thick stoichiometric Si_3_N_4_ film using low-pressure chemical vapor deposition on top the lower cladding thermal oxide. The waveguides are patterned using a standard deep ultraviolet (DUV) stepper on the DUV photoresist layer that was spun on the waveguide layer. The waveguide core is patterned by dry etching the Si_3_N_4_ layer anisotropically using an inductively coupled plasma etcher with a CHF_3_/CF_4_/O_2_ chemistry. This is followed by a cleaning step with a standard Radio Corporation of America process. A two-step (3 µm each) plasma-enhanced chemical vapor deposition (PECVD) with tetraethoxysilane (TEOS) as a precursor is used to form the 6 µm-thick silicon dioxide upper cladding. The final step in fabrication is a two-step anneal at 1050 °C for 7 h and 1150 °C for 2 h, which is optimized anneal process for our waveguides.

### Multi-physics simulations of Brillouin scattering

To simulate the Brillouin gain spectrum, we use finite element solvers to obtain the optical modes of the SBS laser resonator and then use these modes to construct electrostrictive forces, and determine the mechanical response of the system to these time-harmonic forces. Using an argument based on the Manley–Rowe relations, we obtain the Brillouin gain (${G}_{{{{{{\rm{B}}}}}}}\left(\varOmega \right)$) from the dissipated mechanical power when the system is driven by electrostrictive forces:

1 $${G}_{{{{{{\rm{B}}}}}}}=\frac{1}{\delta z}\frac{{\omega }_{{{{{{\rm{S}}}}}}}}{\varOmega }\frac{1}{{P}_{{{{{{\rm{p}}}}}}}{P}_{{{{{{\rm{S}}}}}}}}\int {d}^{3}x\left\langle {{{{{\bf{f}}}}}}\cdot \dot{{{{{{\bf{u}}}}}}}\right\rangle$$

where ${\omega }_{{{{{{\rm{S}}}}}}}$ and $\varOmega$ are the angular frequencies of the Stokes and phonon modes respectively, $\delta z$ is the length of the waveguide, ${P}_{{{{{{\rm{p}}}}}}}({P}_{{{{{{\rm{S}}}}}}})$ is the power in the pump (Stokes) modes used to construct the optical forces, ${{{{{\bf{u}}}}}}$ is the elastic displacement, and ${{{{{\bf{f}}}}}}$ is the electrostrictive force density. The dissipated mechanical power is represented here as the volume integral of the time-averaged power $\int {d}^{3}x\left\langle {{{{{\bf{f}}}}}}\cdot \dot{{{{{{\bf{u}}}}}}}\right\rangle .$ To obtain the electrostrictive force, the photoelastic tensor (${p}_{{ijkl}}$) and the optical mode components are combined to give the *i*_th_ component of the force density given by

2 $${f}_{i}=\frac{1}{4}{\epsilon }_{0}{\partial }_{j}{n}^{4}{p}_{{ijkl}}({E}_{p,k}{E}_{S,l}^{\ast }+{E}_{p,l}{E}_{S,k}^{\ast })$$

where ${E}_{p,k}$ and ${E}_{S,k}$ are the *k*^*th*^ components of the pump and Stokes electric fields, respectively. In addition to providing the magnitude, bandwidth and peak of the Brillouin gain these simulations provide insights about the spectrum structure. For example, our simulations show that phonon interference with the air-cladding boundary explains the modulation of the gain spectrum with frequency shown in Fig. 2c.

### Resonator linewidth measurements

To measure the 674 nm resonator we use a commercially available external cat-eye diode tunable pump laser (from MOGLabs^TM^). For calibrating the resonator linewidth, we use a fiber based (external) ~50 meter unbalanced radio frequency (RF) calibrated MZI. The FSR of the MZI is calibrated using an RF electro-optic phase modulator (EOM) whose sidebands scan across the resonance. The measured MZI FSR is 3.99 ± 0.02 MHz. The MZI is acoustically isolated to minimize noise in the fringes. A small portion (1%) of the laser power is tapped and sent to the MZI to simultaneously provide the RF calibrated frequency references.

For the 698 nm resonator, we use a Ti:Sapphire laser at 698 nm. To calibrate the frequency, we use two different phase modulators to add sidebands at 1.4645 GHz for FSR measurements and at 60 MHz for Q measurements.

### Frequency noise measurements

Measurement of the frequency noise and fundamental linewidth are performed using an OFD whose two parts are a fiber based unbalanced MZI (~50 m fiber length, ~3.99 MHz FSR) and a balanced photodetector (BPD, Thorlabs PDB450A). The relation between the detector’s output power spectral density, ${S}_{{{{{{\rm{out}}}}}}}\left(\nu \right)$ in (V^2^Hz^−1^) and the laser’s frequency noise, ${S}_{{{{{{\rm{f}}}}}}}\left(\nu \right)$ in (Hz^2^Hz^−1^) is given by:

3 $${S}_{{{{{{\rm{f}}}}}}}\left(\nu \right)={S}_{{{{{{\rm{out}}}}}}}\left(\nu \right){\left(\frac{\nu }{{{{{{\rm{sin }}}}}}({{\pi }}\nu {\tau }_{{{{{{\rm{D}}}}}}}){V}_{{{{{{\rm{PP}}}}}}}}\right)}^{2}$$

where ${\tau }_{{{{{{\rm{D}}}}}}}$ is the MZI’s optical delay, $\nu$ is the frequency offset. The detector’s output peak to peak voltage is ${V}_{{{{{{\rm{PP}}}}}}}$. A tangent is taken at the lowest point of the ${S}_{{{{{{\rm{f}}}}}}}\left(\nu \right)$, usually far-from-carrier (>1 MHz), to give the value ${S}_{{{{{{\rm{w}}}}}}}$. The Fundamental linewidth is given by $\Delta \nu ={{{{{\rm{\pi }}}}}}{S}_{{{{{{\rm{w}}}}}}}$.

The S1 power is collected from the circulator reflection port and is sent into the unbalanced MZI which is housed in an enclosure to isolate from acoustic disturbances. The two MZI outputs are inputs of the two arms of the BPD. The bandwidth of the BPD is set to its highest value of 150 MHz to minimize the impact of intensity variations in the BPD output. The power spectral density ${S}_{{{{{{\rm{out}}}}}}}\left(\nu \right)$ of the BPD RF (difference) output is measured using a digital sampling oscilloscope (Keysight DSOX1204G with a 200 MHz bandwidth). The RF output triggers the scope at the MZI’s quadrature operating point and the power spectral density data are averaged over 16 traces with a Hann window applied. The frequency noise is calculated using Eq. (3).

### Reporting summary

Further information on research design is available in the Nature Research Reporting Summary linked to this article.

## Supplementary information

Supplementary Information

Reporting Summary

## Data Availability

The data that support the plots within this paper and other findings of this study are available from the corresponding author on reasonable request.

## References

[1] Ludlow AD, Boyd MM, Ye J, Peik E, Schmidt PO (2015). Optical atomic clocks. Rev. Mod. Phys..

[2] Young AW (2020). Half-minute-scale atomic coherence and high relative stability in a tweezer clock. Nature.

[3] Cygan A (2013). Cavity mode-width spectroscopy with widely tunable ultra narrow laser. Opt. Express, OE.

[4] Jadbabaie, A., Pilgram, N. H., Klos, J., Kotochigova, S. & Hutzler, N. R. Enhanced molecular yield from a cryogenic buffer gas beam source via excited state chemistry. *New J. Phys*. 10.1088/1367-2630/ab6eae (2020).

[5] Bohn JL, Rey AM, Ye J (2017). Cold molecules: progress in quantum engineering of chemistry and quantum matter. Science.

[6] Degen CL, Reinhard F, Cappellaro P (2017). Quantum sensing. Rev. Mod. Phys..

[7] Fan H (2015). Atom based RF electric field sensing. J. Phys. B: . Mol. Opt. Phys..

[8] Fox, R. W., Oates, C. W. & Hollberg, L. W. 1. Stabilizing diode lasers to high-finesse cavities. in *Experimental Methods in the Physical Sciences* (eds. van Zee, R. D. & Looney, J. P.) vol. 40 1–46 (Academic Press, 2003).

[9] Ludlow AD (2007). Compact, thermal-noise-limited optical cavity for diode laser stabilization at 1×10^−15^. Opt. Lett., OL.

[10] Ludlow, A. D. The Strontium Optical Lattice Clock: optical Spectroscopy with Sub-Hertz Accuracy. http://meetings.aps.org/link/BAPS.2009.DAMOP.C1.2 (2009).

[11] Nicholson TL (2015). Systematic evaluation of an atomic clock at 2 × 10−18 total uncertainty. Nat. Commun..

[12] Blumenthal DJ, Heideman R, Geuzebroek D, Leinse A, Roeloffzen C (2018). Silicon Nitride in Silicon Photonics. Proc. IEEE.

[13] Blumenthal DJ (2020). Photonic integration for UV to IR applications. APL Photon..

[14] Liu AY, Bowers J (2018). Photonic Integration With Epitaxial III–V on Silicon. IEEE J. Sel. Top. Quantum Electron..

[15] Bloch I (2008). Quantum coherence and entanglement with ultracold atoms in optical lattices. Nature.

[16] Levine H (2018). High-Fidelity Control and Entanglement of Rydberg-Atom Qubits. Phys. Rev. Lett..

[17] Hutzler NR (2020). Polyatomic molecules as quantum sensors for fundamental physics. Quantum Sci. Technol..

[18] Krawinkel, T. Improved GNSS navigation with chip-scale atomic clocks. *Veröffentlichungen der DGK, Reihe C, Dissertationen*. **823**, 10.15488/4684 (2018).

[19] Ospelkaus S (2010). Quantum-State Controlled Chemical Reactions of Ultracold Potassium-Rubidium Molecules. Science.

[20] National Academies of Sciences, E. *Manipulating Quantum Systems: An Assessment of Atomic, Molecular, and Optical Physics in the United States*. 10.17226/25613 (2019).

[21] Yu P, Hutzler NR (2021). Probing Fundamental Symmetries of Deformed Nuclei in Symmetric Top Molecules. Phys. Rev. Lett..

[22] Verma M, Jayich AM, Vutha AC (2020). Electron Electric Dipole Moment Searches Using Clock Transitions in Ultracold Molecules. Phys. Rev. Lett..

[23] Anderegg L (2019). An optical tweezer array of ultracold molecules. Science.

[24] Mitra D (2020). Direct laser cooling of a symmetric top molecule. Science.

[25] Debut A, Randoux S, Zemmouri J (2000). Linewidth narrowing in Brillouin lasers: theoretical analysis. Phys. Rev. A.

[26] Woodward RI, Kelleher EJR, Popov SV, Taylor JR (2014). Stimulated Brillouin scattering of visible light in small-core photonic crystal fibers. Opt. Lett., OL.

[27] Ponikvar DR, Ezekiel S (1981). Stabilized single-frequency stimulated Brillouin fiber ring laser. Opt. Lett., OL.

[28] Smith SP, Zarinetchi F, Ezekiel S (1991). Narrow-linewidth stimulated Brillouin fiber laser and applications. Opt. Lett., OL.

[29] Zarinetchi F, Smith SP, Ezekiel S (1991). Stimulated Brillouin fiber-optic laser gyroscope. Opt. Lett., OL.

[30] Stokes LF, Chodorow M, Shaw HJ (1982). All-fiber stimulated Brillouin ring laser with submilliwatt pump threshold. Opt. Lett., OL.

[31] Bai Z (2020). Diamond Brillouin laser in the visible. APL Photon..

[32] Jiang S (2019). Visible Raman and Brillouin lasers from a microresonator/ZBLAN-fiber hybrid system. Photon. Res., PRJ.

[33] Loh W (2020). Operation of an optical atomic clock with a Brillouin laser subsystem. Nature.

[34] Gundavarapu S (2019). Sub-hertz fundamental linewidth photonic integrated Brillouin laser. Nat. Photon..

[35] Rakich PT, Reinke C, Camacho R, Davids P, Wang Z (2012). Giant Enhancement of Stimulated Brillouin Scattering in the Subwavelength Limit. Phys. Rev. X.

[36] Pant R (2011). On-chip stimulated Brillouin scattering. Opt. Express, OE.

[37] Poulton CG, Pant R, Eggleton BJ (2013). Acoustic confinement and stimulated Brillouin scattering in integrated optical waveguides. J. Opt. Soc. Am. B, JOSAB.

[38] Li J, Lee H, Chen T, Vahala KJ (2012). Characterization of a high coherence, Brillouin microcavity laser on silicon. Opt. Express, OE.

[39] Yang KY (2018). Bridging ultrahigh- Q devices and photonic circuits. Nat. Photon..

[40] Eggleton BJ, Poulton CG, Rakich PT, Steel MJ, Bahl G (2019). Brillouin integrated photonics. Nat. Photon..

[41] Gyger F (2020). Observation of Stimulated Brillouin Scattering in Silicon Nitride Integrated Waveguides. Phys. Rev. Lett..

[42] Brodnik, G. M. et al. Chip-Scale, Optical-Frequency-Stabilized PLL for DSP-Free, Low-Power Coherent QAM in the DCI. in *Optical Fiber Communication Conference (OFC) 2020 (2020), paper M3A.6* M3A.6 (Optical Society of America, 2020). 10.1364/OFC.2020.M3A.6.

[43] Chauhan, N., Wang, J., Bose, D., Moreira, R. & Blumenthal, D. J. Ultra-Low Loss 698 nm and 450 nm Silicon Nitride Visible Wavelength Waveguides for Strontium Atomic Clock Applications. in *Conference on Lasers and Electro-Optics* STh1J.2 (OSA, 2020). 10.1364/CLEO_SI.2020.STh1J.2.

[44] Lin, Y. et al. Narrow linewidth hybrid InP-TriPleX photonic integrated tunable laser based on silicon nitride micro-ring resonators. in *Optical Fiber Communication Conference* Th2A.14 (OSA, 2018). 10.1364/OFC.2018.Th2A.14.

[45] Bramhavar, S. et al. A visible-light integrated photonic platform for atomic systems. in *Integrated Optics: Devices, Materials, and Technologies XXIII* vol. 10921 109211D (International Society for Optics and Photonics, 2019).

[46] Park H, Zhang C, Tran MA, Komljenovic T (2020). Heterogeneous silicon nitride photonics. Opt., Opt..

[47] Huffman TA (2018). Integrated Resonators in an Ultralow Loss Si_3_N_4_/SiO_2_ Platform for Multifunction Applications. IEEE J. Sel. Top. Quantum Electron..

[48] Kittlaus EA (2021). Electrically driven acousto-optics and broadband non-reciprocity in silicon photonics. Nat. Photon..

[49] Shao L (2020). Integrated microwave acousto-optic frequency shifter on thin-film lithium niobate. Opt. Express, OE.

[50] Zhao Q (2020). Low-loss low thermo-optic coefficient Ta2O5 on crystal quartz planar optical waveguides. APL Photon..

[51] Lee H (2013). Spiral resonators for on-chip laser frequency stabilization. Nat. Commun..

[52] Ghosh, P. K. Ion traps. 6–46 (Clarendon Press, 1995).

[53] Niffenegger RJ (2020). Integrated multi-wavelength control of an ion qubit. Nature.

[54] Chauhan, N. et al. Photonic Integrated Si3N4 Ultra-Large-Area Grating Waveguide MOT Interface for 3D Atomic Clock Laser Cooling. in *Conference on Lasers and Electro-Optics* STu4O.3 (OSA, 2019). 10.1364/CLEO_SI.2019.STu4O.3.

[55] Hummon MT (2018). Photonic chip for laser stabilization to an atomic vapor with 10 ^−11^ instability. Optica.

[56] Behunin RO, Otterstrom NT, Rakich PT, Gundavarapu S, Blumenthal DJ (2018). Fundamental noise dynamics in cascaded-order Brillouin lasers. Phys. Rev. A.

[57] Kozlov VG, Bulović V, Burrows PE, Forrest SR (1997). Laser action in organic semiconductor waveguide and double-heterostructure devices. Nature.

[58] Lee BS (2017). On-chip thermo-optic tuning of suspended microresonators. Opt. Express, OE.

[59] Wang, J. et al. Fully symmetric controllable integrated three-resonator photonic molecule. *arXiv:2105.10815 [physics]* (2021).

[60] Puckett MW (2021). 422 Million intrinsic quality factor planar integrated all-waveguide resonator with sub-MHz linewidth. Nat. Commun..

[61] Clark, S. M. et al. Engineering the Quantum Scientific Computing Open User Testbed (QSCOUT): design details and user guide. *arXiv:2104.00759 [quant-ph]* (2021).

[62] Lischke, S. et al. Silicon nitride waveguide coupled 67+ GHz Ge photodiode for non-SOI PIC and ePIC platforms. in *2019 IEEE International Electron Devices Meeting (IEDM)* 33.2.1–33.2.4 10.1109/IEDM19573.2019.8993651 (2019).

