# Supplementary material for: Visible light photonic integrated Brillouin laser
Source: Nat Commun. 2021 Aug 3;12:4685. doi: 10.1038/s41467-021-24926-8 (PMC8333255; doi:10.1038/s41467-021-24926-8)
Supplement: Supplementary file 1 — Supplementary Information [file 41467_2021_24926_MOESM1_ESM.pdf]

Supplementary Information

Visible light photonic integrated Brillouin laser

Nitesh Chauhan[1], Andrei Isichenko[1], Kaikai Liu[1], Jiawei Wang[1], Qiancheng Zhao[1], Ryan O. Behunin[2,3], Peter T. Rakich[4], Andrew M. Jayich[5], C. Fertig[6], C. W. Hoyt[6], Daniel J. Blumenthal[1*]
[1]Department of Electrical and Computer Engineering, University of California Santa Barbara, Santa Barbara, CA, USA.
[2]Department of Applied Physics and Materials Science, Northern Arizona University, Flagstaff, AZ, USA.
[3]Center for Materials Interfaces in Research and Applications, Northern Arizona University, Flagstaff, AZ, USA
[4]Department of Applied Physics, Yale University, New Haven, CT, USA
[5]Department Physics, University of California Santa Barbara, Santa Barbara, CA, USA.
[6]Honeywell International, Plymouth, MN, USA.

*Corresponding author (danb@ucsb.edu)

Supplementary Note 1: Introduction

In this Supplementary Information, we reveal more details on the measurement setup for the spontaneous and stimulated Brillouin measurement; we discuss in detail the modelling of stokes power as the on chip power is increased above threshold.

Supplementary Note 2: Spontaneous Brillouin scattering measurement setup

Spontaneous Brillouin scattering is scattering by uncorrelated incoherent photon-phonon gratings resulting in very weak backscattered signals. To reliably measure the spontaneous backscattering signal a 40 dB gain radio frequency (RF) amplifier is used to boost the photodiode (PD) signal. The resolution bandwidth (RBW) of the electrical spectrum analyzer is set to 100 Hz to increase the sensitivity. 20 traces are averaged on the ESA to reduce the noise. A background trace is taken with no input to PD and all settings of ESA kept the same and subtracted from the measurements to remove instrument background noise. Traces are taken with device coupled and that yields Brillouin scattered signal from waveguide (2 m spiral) as well as ~ 0.5 m fiber between circulator and device facet. Another measurement is taken with the device uncoupled to identify the peak from the fiber section while keeping the power constant. The fiber peak remains unchanged while the waveguide peak disappears. The setup schematic is shown in the Supplementary Figure 1.

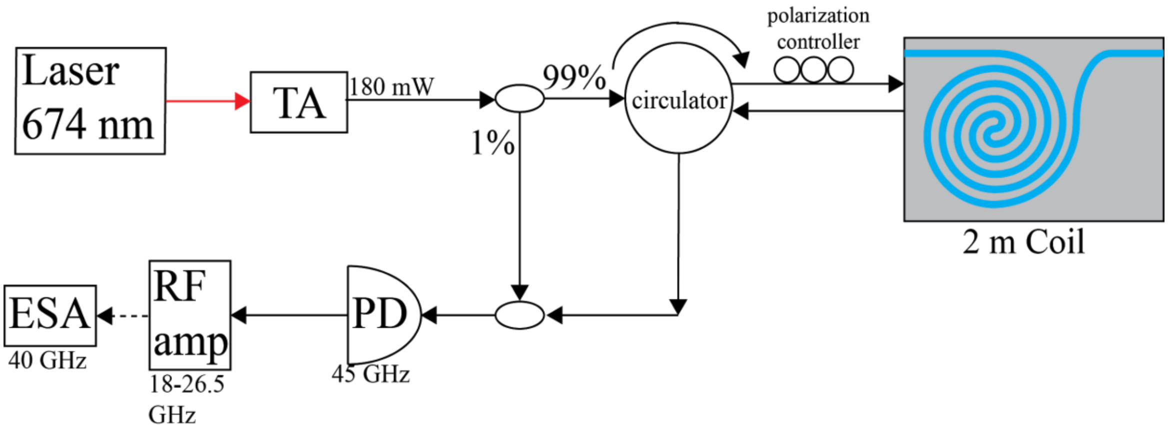

Supplementary Figure 1. Setup schematic for spontaneous Brillouin scattering spectrum measurement. Setup for measuring spontaneous Brillouin scattering. The 674 nm laser power is amplified with a tapered amplifier (TA), 1% of power is tapped out to beat with the Stokes tone which is obtained from the circulator. A high bandwidth 45 GHz photodiode (PD) is used to detect the 25.11 GHz stokes tone and is amplified using a large 40 dB gain radio frequency amplifier onto a 40 GHz electric spectrum analyzer (ESA).

Supplementary Note 3: SBS lasing measurement setup

The measurements of Brillouin gain spectrum before and after threshold are made with the same setup as for spontaneous Brillouin measurements shown in Supplementary Figure 1 with the only difference being the laser being locked to a commercially available high finesse (Stable Laser System^{TM}; 150,000 finesse), low thermal expansion stable cavity and the rf amp is not used after PD since the resonance enhanced spontaneous and the stimulated Brillouin signal is much stronger than spontaneous signal from the spiral. The laser was locked to stable cavity to avoid pump noise from affecting heterodyne beat note (convolution of pump and Stokes) on the ESA. This is the only measurement in which the pump was locked. The RBW is set to 1 kHz.

For the frequency noise measurements, we use an optical frequency discriminator (OFD) consisting of a fiber based unbalanced MZI (UMZI) and a balanced photodetector as shown in Supplementary Figure 2. The laser is not locked to any stable cavity for FN measurements and is not Pound-Drever-Hall (PDH) locked to the resonator; therefore, the noise suppression comes from the SBS phase noise suppression only. The device was on a temperature stage with 0.1 mK resolution to tune to and stabilize the resonance (Vescent^{TM} Slice QT).

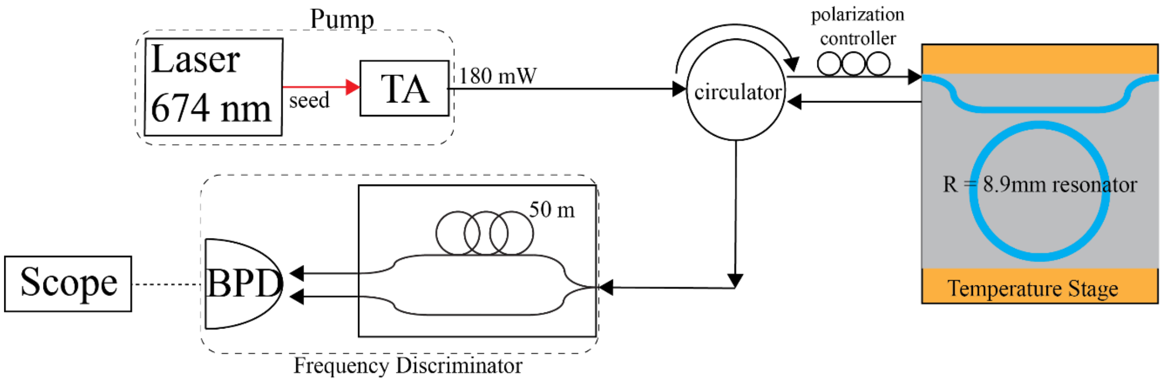

Supplementary Figure 2. Setup schematic for SBS lasing Stokes power and FN measurement. Setup for measuring frequency noise (FN). Details of frequency discriminator used for measurement in methods section.

698 nm clock SBS laser measurements. The apparatus for the initial demonstration of a photonic integrated SBS laser at 698 nm is shown in Supplementary Figure 3. A continuous-wave Ti:sapphire laser is tuned near the clock wavelength. At 698.4 nm, the maximum output power in free space is approximately 700 mW. Pump light is coupled through an optical fiber to the input of the bus waveguide. Light polarization is adjusted in the fiber to transverse-electric (TE) for maximum coupling to the resonator. As shown in Supplementary Figure 3, backward-propagating light is directed to an OSA and an ESA using a quarter waveplate and polarizing beam splitter. The light propagating through the bus waveguide to the other side of the photonic chip is measured with a photodetector to monitor the resonance condition. The OSA is a scanning Fabry-Perot spectrometer (Bristol Instruments) that functions as a wavemeter and spectrum analyzer with ~5 GHz resolution. A photodetector (Thorlabs, 25 GHz 3dB bandwidth) converts the SBS laser light to an electrical signal through a beat note with the pump as shown in Supplementary Figure 3. The temperature of the photonic chip is maintained at +/- 0.01 °C by using a thermo-electric cooler.

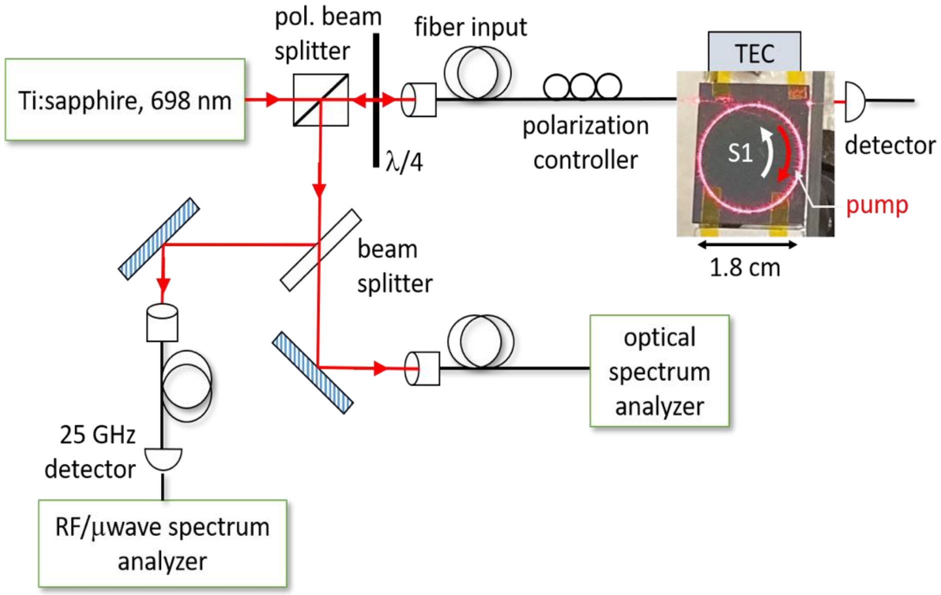

Supplementary Figure 3. Setup schematic for SBS lasing Stokes power measurement. Setup for measuring Stokes power and Brillouin frequency shift in resonator at 698 nm. A thermo electric coupler (TEC) is used to tune and stabilize the resonance.

Supplementary Note 4: Waveguide mode profiles at 674 nm and 698 nm

The waveguides are designed to support a single transverse electric (TE) mode in low loss, high aspect ratio Si_3N_4 waveguide[1]. The waveguide cross section and mode profiles of the TE mode are plotted in Supplementary Figure 4. The same waveguide geometry shown in Supplementary Figure 4**a** supports a TE mode at both 674 nm and 698 nm wavelengths. The bend loss is negligible for our resonator designs. The mode areas in our dilute low index contrast waveguides is 3.0 μm at 674 nm and 3.3 μm at 698 nm. This means that same waveguide supports SBS operation at both wavelengths by just changing ring radius and coupling gaps. Same core height can also be used for different wavelengths but changing the width to support single TE mode. The width, ring radius and coupling gap are all mask level changes.

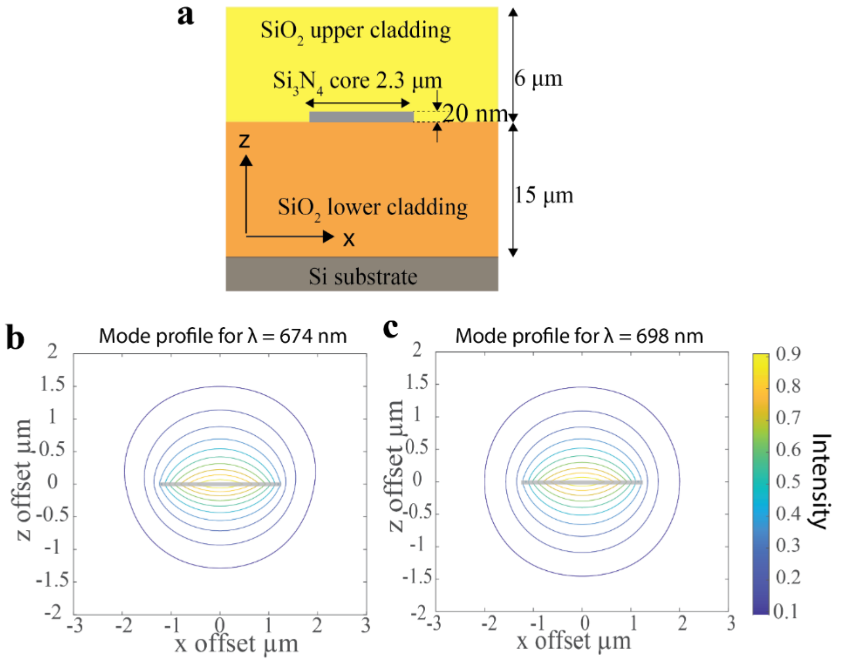

Supplementary Figure 4. Waveguide cross section and mode profiles at 674 nm and 698 nm. a, Waveguide cross section with dimensions. **b,** Contour plot of simulated mode profile at 674 nm, waveguide represented in grey at center. **c,** Contour plot of simulated mode profile at 674 nm, waveguide represented in grey at center.

Supplementary Note 5: Multi-Physics Simulation

To simulate the Brillouin gain spectrum, we use finite element solvers to obtain the optical modes of the SBS laser resonator and then use these modes to construct electrostrictive forces, and determine the mechanical response of the system to these time-harmonic forces. Using an

argument based on the Manley-Rowe relations, we obtain the Brillouin gain ($G_B(\Omega)$) from the dissipated mechanical power when the system is driven by electrostrictive forces[3]:

$$G_B = \frac{1}{\delta z}\frac{\omega_S}{\Omega}\frac{1}{P_p P_S}\int d^3x\langle \mathbf{f}\cdot\dot{\mathbf{u}}\rangle \tag{1}$$

where ω_S and Ω are the angular frequencies of the Stokes and phonon modes respectively, δz is the length of the waveguide, P_p (P_S) is the power in the pump (Stokes) modes used to construct the optical forces, $\mathbf{u}$ is the elastic displacement, and $\mathbf{f}$ is the electrostrictive force density. The dissipated mechanical power is represented here as the volume integral of the time-averaged power $\int d^3x\langle \boldsymbol{f}\cdot\dot{\boldsymbol{u}}\rangle$. To obtain the electrostrictive force, the photoelastic tensor (p_{ijkl}) and the optical mode components are combined to give the i_{th} component of the force density given by

$$f_i = \frac{1}{4}\epsilon_0\partial_j n^4 p_{ijkl}(E_{p,k}E^*_{S,l} + E_{p,l}E^*_{S,k}) \tag{2}$$

where $E_{p,k}$ and $E_{S,k}$ are the kth components of the pump and Stokes electric fields, respectively[4]. The guided optical mode is shown in Supplementary Figure 5**a** and the continuum of non-guided acoustic mode is shown in Supplementary Figure 5**b**. The non-guided acoustic mode is responsible for asymmetric shape of gain currve[5]. In addition to providing the magnitude, bandwidth and peak of the Brillouin gain these simulations provide insights about the spectrum structure. For example, our simulations show that phonon interference with the air-cladding boundary explains the modulation of the gain spectrum with frequency shown in Supplementary Figure 5**c**.

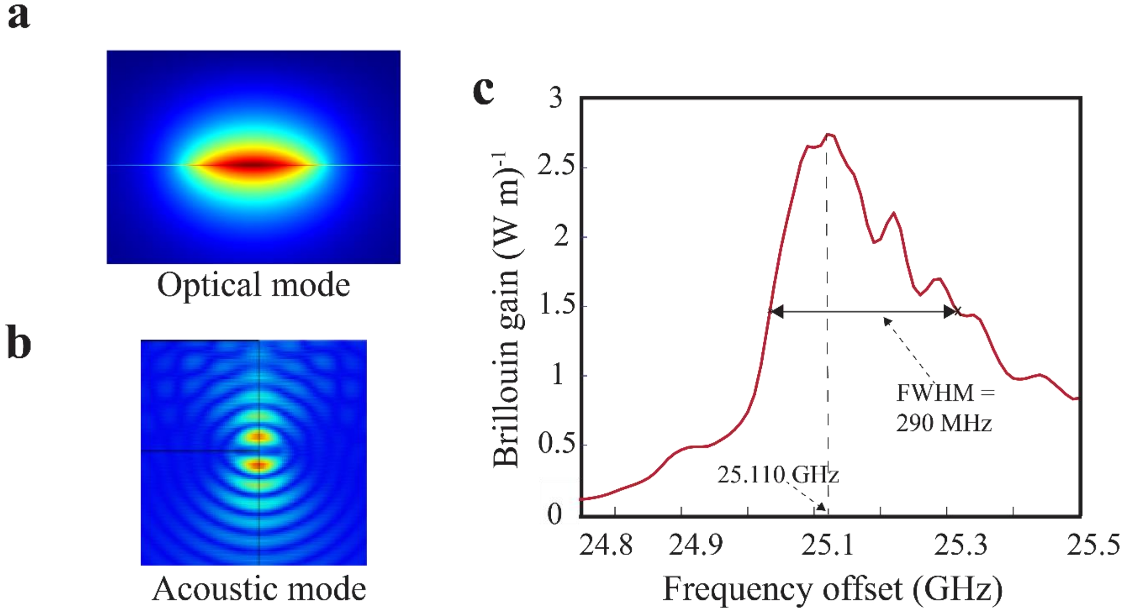

Supplementary Figure 5. Brillouin gain simulation. a, Mode profile of optical mode in waveguide. **b,** Continuum of non guided acoustic modes **c.** Simulated spontaneous Brillouin scattering spectrum.

Supplementary Note 6: SBS lasing Stokes power modelling

We use the simplified coupled mode approach to model the intracavity photon number dynamics and the equations of motion for the optical field amplitude are given by[2]

$$\dot{a}_m = -\left(\frac{\gamma}{2} + \mu|a_m + 1|^2 - \mu|a_m - 1|^2\right) a_m + \sqrt{\gamma_c} F_{pump} \delta_{m0} \tag{3}$$

where the total loss rate, $\gamma = \gamma_{in} + \gamma_c$, is the sum of the intrinsic and coupling loss rates, μ is the Brillouin amplification rate per pump photon, and $F_{pump} = \sqrt{P_{pump}/h\nu}$ is the photon influx field amplitude. The Brillouin amplification rate μ of the SBS resonator is a function of Brillouin gain factor and resonator parameters,

$$\mu = \frac{h\nu v_g^2 G_{\mathrm{B}}}{2L} \tag{4}$$

Where $G_{\mathrm{B}} = g_{\mathrm{B}}/A_{\mathrm{eff}}$ is the Brillouin gain factor, g_{B} is the bulk Brillouin gain and L is the resonator roundtrip length. From Supplementary Equation 3, we can find the threshold power for S1,

$$P_{\mathrm{th}} = \frac{h\nu\gamma^3}{8\mu\gamma_{\mathrm{c}}} \tag{5}$$

and the threshold power for S2 and S3 are $4P_{\mathrm{th}}$ and $8P_{\mathrm{th}}$.
The fundamental linewidth for S1 before its clamping is found to be,

$$\delta\nu_1 = \frac{n_0\gamma}{4\pi|a_1|^2} \tag{6}$$

and it reaches its minimum at the S1 clamping point,

$$\delta\nu_1(4P_{\mathrm{th}}) = \frac{n_0\mu}{2\pi} \tag{7}$$

where $n_0 = k_m T/(\hbar\Omega)$ is the thermal occupation numbers of the acoustic mode. For the acoustic frequency 25.110 GHz, n_0 at room temperature is estimated to be ~1300. From the measured on-chip threshold power of 14.7 mW, the resonator intrinsic linewidth of 8.0 MHz, the resonator coupling linewidth of 8.0 MHz, the resonator mode area of 3.0 μm^2 and the ring radius of 8.95 mm, we estimate the Brillouin gain rate μ to be 50.7 mHz (the corresponding Brillouin gain GB of 0.49 (W m)$^{-1}$) using Supplementary Equation 4 and Supplementary Equation 5 and the minimal fundamental linewidth of S1 to be 2.0 Hz using Supplementary Equation 6, shown in Supplementary Figure 6. This estimated Brillouin gain GB of 0.49 (W m)$^{-1}$ is close to the simulated GB of 1.03 (W m)$^{-1}$ at the Brillouin shift frequency of 25.036 GHz (imperfectly aligned FSR from the Brillouin shift of 25.11 GHz) from the full COMSOL optomechanics simulation shown in Supplementary Figure 5**c**, showing a rough agreement between experiment and physics simulation.

The theoretical S1 threshold of 7.0 mW assuming our simulated G_B of 1.03 $(\mathrm{W\ m})^{-1}$ is also close to the measured threshold of 14.7 mW.

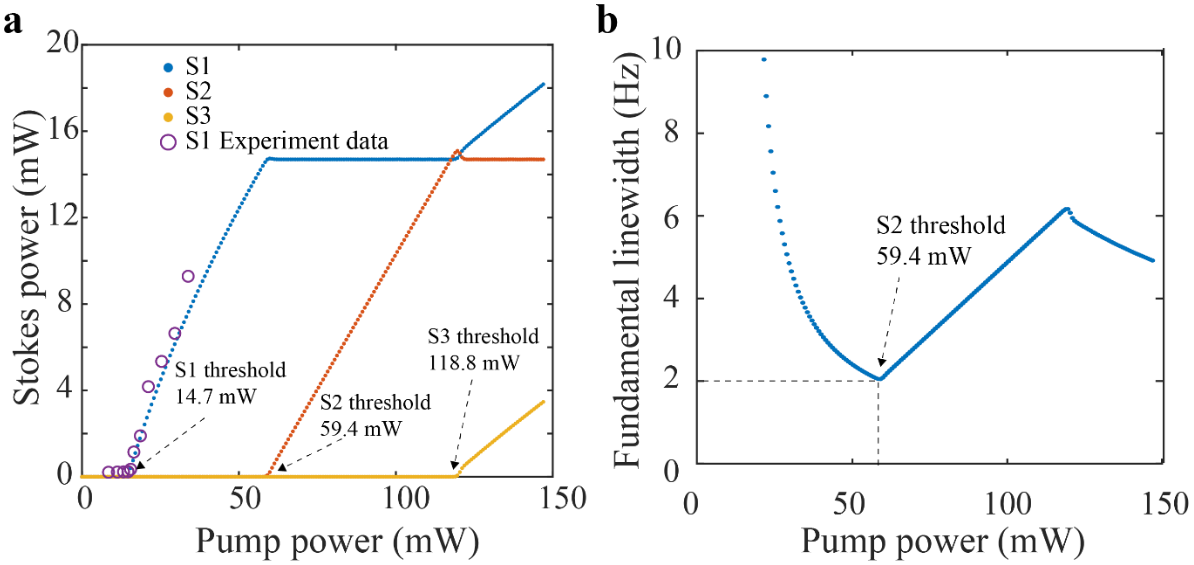

Supplementary Figure 6. Setup schematic for SBS lasing Stokes power measurement and modelling. a, Modeled Stokes power for on chip power in range of 0-150 mW. **b,** Simulated fundamental linewidth of first stokes tone as a function of on-chip power assuming no crosstalk from pump. The fundamental linewidth decreases until S2 threshold is reached.

Discussion on slope Efficiency: Here we are defining the slope efficiency as the fraction of pump photons that are converted to Stokes photons above threshold. By modeling the laser emission, we find the conversion efficiency, defined as the ratio between the S1 output power and the pump power, is:

$$\eta = 2\left(\frac{\gamma_{\mathrm{c}}}{\gamma}\right)^2 \tag{8}$$

where γ_{c} is the bus-ring coupling loss rate and $\gamma = \gamma_{\mathrm{in}} + \gamma_{\mathrm{c}}$ is the total loss rate (sum of the coupling rate and waveguide loss rate). This means that the slope efficiency is 50% in critically coupled resonators, and any deviation from critically coupled towards undercoupled ($\gamma_{in} > \gamma_c$) will decrease the slope efficiency for all pass ring resonators like ours. The slope efficiency can be increased above 50% by designing overcoupled resonators ($\gamma_{in} < \gamma_c$) at the cost of increased threshold power (Supplementary Equation 5) for lasing. Thus, we chose critically coupled resonators as a compromise between the threshold and slope efficiency for SBS lasing in an all pass resonator.

Supplementary References

1. Chauhan, N., Wang, J., Bose, D., Moreira, R. & Blumenthal, D. J. Ultra-Low Loss 698 nm and 450 nm Silicon Nitride Visible Wavelength Waveguides for Strontium Atomic Clock Applications. in *Conference on Lasers and Electro-Optics* STh1J.2 (OSA, 2020). doi:10.1364/CLEO_SI.2020.STh1J.2.
2. Behunin, R. O., Otterstrom, N. T., Rakich, P. T., Gundavarapu, S. & Blumenthal, D. J. Fundamental noise dynamics in cascaded-order Brillouin lasers. *Phys. Rev. A* **98**, 023832 (2018).
3. Rakich, P. T., Reinke, C., Camacho, R., Davids, P. & Wang, Z. Giant Enhancement of Stimulated Brillouin Scattering in the Subwavelength Limit. *Phys. Rev. X* **2**, 011008 (2012).
4. Qiu, W. *et al.* Stimulated Brillouin scattering in nanoscale silicon step-index waveguides: a general framework of selection rules and calculating SBS gain. *Opt. Express, OE* **21**, 31402–31419 (2013).
5. Poulton, C. G., Pant, R. & Eggleton, B. J. Acoustic confinement and stimulated Brillouin scattering in integrated optical waveguides. *J. Opt. Soc. Am. B* **30**, 2657 (2013).
