# Supplementary material for: Visible light photonic integrated Brillouin laser
Source: Nat Commun. 2021 Aug 3;12:4685. doi: 10.1038/s41467-021-24926-8 (PMC8333255; doi:10.1038/s41467-021-24926-8)
Supplement: Supplementary file 2 — Reporting Summary [file 41467_2021_24926_MOESM2_ESM.pdf]

nature research

Corresponding author(s): Daniel J. Blumenthal NCOMMS-21-08770

Lasing Reporting Summary

Nature Research wishes to improve the reproducibility of the work that we publish. This form is intended for publication with all accepted papers reporting claims of lasing and provides structure for consistency and transparency in reporting. Some list items might not apply to an individual manuscript, but all fields must be completed for clarity.

For further information on Nature Research policies, including our data availability policy, see Authors & Referees.

Experimental design

Please check: are the following details reported in the manuscript?

1. Threshold

Plots of device output power versus pump power over a wide range of values indicating a clear threshold

[x] Yes
[ ] No

We have demonstrated threshold by varying input pump power and measuring power in the first order Stokes (S1). Main results see: Visible light 674 nm SBS lasing section and Fig. 4a.

2. Linewidth narrowing

Plots of spectral power density for the emission at pump powers below, around, and above the lasing threshold, indicating a clear linewidth narrowing at threshold

[x] Yes
[ ] No

We have demonstrated linewidth narrowing as the input pump power is increased, from below threshold, just below and just above threshold, and above threshold. With the pump power below, just below and just above threshold, we measured the S1 linewidth using a heterodyne beatnote and an electrical spectrum analyzer, demonstarting that below threshold, the linewidth is the passive resonator linewidth filtering the sponteneous Brillouin. Then, approaching and just above threhold, the S1 linewidth as measured on the ESA collapses to 120 KHz, an order of magnitude less than the passive cavity linewidth, demonstrating that we have entered the stimulated Brillouin regime. To measure the linewidth narrow than the capablity of the beatnote and ESA, we then measure the frequency noise using an optical frequency noise discriminator, and demonstrate that the fundamental linewidth continues to reduce (far from carrier noise) to under 300 Hz as the pump continues to increase past threshold. See main text: Visible light 674 nm SBS lasing section, and Fig. 4b,c,d.

Resolution of the spectrometer used to make spectral measurements

[x] Yes
[ ] No

Spontaneous measurement RBW 100 Hz, span 1 GHz, SBS measurements RBW 1 kHz span 50MHz; scan speed determined by RBW, not independent parameter. See main text Fig 2c, Fig. 4b

3. Coherent emission

Measurements of the coherence and/or polarization of the emission

[x] Yes
[ ] No

Main text results : Visible light 674 nm SBS lasing and Fig. 4c,d.

4. Beam spatial profile

Image and/or measurement of the spatial shape and profile of the emission, showing a well-defined beam above threshold

[ ] Yes
[x] No

NA. Photonic integrated waveguide device and emission.

5. Operating conditions

Description of the laser and pumping conditions
Continuous-wave, pulsed, temperature of operation

[x] Yes
[ ] No

Continuous wave (cw) pump laser @ 674 nm. Operating temperature 25 degrees C. Main text results : Visible light 674 nm SBS lasing and Supplementary note 3

Threshold values provided as density values (e.g. W cm^{-2} or J cm^{-2}) taking into account the area of the device

[x] Yes
[ ] No

Threshold power: 4.93 mW cm -2.Main text results : Visible light 674 nm SBS lasing

6. Alternative explanations

Reasoning as to why alternative explanations have been ruled out as responsible for the emission characteristics
e.g. amplified spontaneous, directional scattering; modification of fluorescence spectrum by the cavity

[x] Yes
[ ] No

Emission frequency is at predicted Brillouin first order Stokes frequency shift and emits in direction counter-propagating to pump as expected. As described above, emission linewidth is orders of magnitude lower than cold-cavity resonance linewidth. Photon and phonon lifetimes give predicted and expected Brillouin linewidth narrowing (phase noise reduction) of pump linewidth). Main text results : Visible light 674 nm SBS lasing (Fig. 4c,d) by showing decreasing in linewidth as on chip pump power is increased typical of SBS laser

7. Theoretical analysis

Theoretical analysis that ensures that the experimental values measured are realistic and reasonable
e.g. laser threshold, linewidth, cavity gain-loss, efficiency

☒ Yes ☐ No

Multiphysics analysis performed using measured material properties acurately predicate first order SBS frequency shift and Brillouin gain spectrum and gain bandwidth. See Methods and Supplementary Note 5. This is a full vectorial phonon-photon simulation of the actual waveguide at wavelength of operation. Predicted threshold 7 mW, measured 14.7 mW, predicted fundamental linewidth (FLW) much lower than measured FW due to crosstalk of back reflected pump measured with S1. At threshold measured FWHM LW collapses from 16 MHz (~ resonator linewidth) to 120 kHz, >100x collapse demonstrating onset of lasing and Brillouin linewidth narrowing properties. See Supplementary Note 6

8. Statistics

Number of devices fabricated and tested

☐ Yes ☒ No

We measured several devices for passive Q and cold-cavity performance, but only one device was demonstrated in lasing.

Statistical analysis of the device performance and lifetime (time to failure)

☐ Yes ☒ No

We have statistical analysis of this type of cavity, report in Nature Communications at 1550 nm (Q = 422 Million). But for this work, we did not do a full spectrum and other statistical analysis, rather, our focus was on demonstrating that the laser structure could be designed and operated in the visible, which has never been demonstrated before.
